# Further Development of the Assessment of Military Multitasking Performance: Iterative Reliability Testing

**DOI:** 10.1371/journal.pone.0169104

**Published:** 2017-01-05

**Authors:** Margaret M. Weightman, Karen L. McCulloch, Mary V. Radomski, Marsha Finkelstein, Amy S. Cecchini, Leslie F. Davidson, Kristin J. Heaton, Laurel B. Smith, Matthew R. Scherer

**Affiliations:** 1 Courage Kenny Research Center, Abbott Northwestern Hospital, Minneapolis, Minnesota, United States of America; 2 Division of Physical Therapy, University of North Carolina-Chapel Hill, Chapel Hill, North Carolina, United States of America; 3 Defense and Veterans Brain Injury Center, Fort Bragg, North Carolina, United States of America; 4 Department of Clinical Research and Leadership, George Washington School of Medicine and Health Sciences, Washington, DC, United States of America; 5 United States Army Research Institute of Environmental Medicine, Natick, Massachusetts, United States of America; 6 Boston University School of Public Health, Department of Environmental Health, Boston, Massachusetts, United States of America; 7 Clinical and Rehabilitative Medicine Research Program, Medical Research Materiel Command, Ft Detrick, Maryland, United States of America; University of Glasgow, UNITED KINGDOM

## Abstract

The Assessment of Military Multitasking Performance (AMMP) is a battery of functional dual-tasks and multitasks based on military activities that target known sensorimotor, cognitive, and exertional vulnerabilities after concussion/mild traumatic brain injury (mTBI). The AMMP was developed to help address known limitations in post concussive return to duty assessment and decision making. Once validated, the AMMP is intended for use in combination with other metrics to inform duty-readiness decisions in Active Duty Service Members following concussion. This study used an iterative process of repeated interrater reliability testing and feasibility feedback to drive modifications to the 9 tasks of the original AMMP which resulted in a final version of 6 tasks with metrics that demonstrated clinically acceptable ICCs of > 0.92 (range of 0.92–1.0) for the 3 dual tasks and > 0.87 (range 0.87–1.0) for the metrics of the 3 multitasks. Three metrics involved in recording subject errors across 2 tasks did not achieve ICCs above 0.85 set apriori for multitasks (0.64) and above 0.90 set for dual-tasks (0.77 and 0.86) and were not used for further analysis. This iterative process involved 3 phases of testing with between 13 and 26 subjects, ages 18–42 years, tested in each phase from a combined cohort of healthy controls and Service Members with mTBI. Study findings support continued validation of this assessment tool to provide rehabilitation clinicians further return to duty assessment methods robust to ceiling effects with strong face validity to injured Warriors and their leaders.

## Introduction

Combat-related exposures, routine operational and training activities as well as common sports and leisure activities all put military service members (SM) at increased risk for sustaining a traumatic brain injury (TBI) in both theater and garrison environments. In excess of 350,000 Service Members (SM) sustained at least one TBI from 2000 through the middle of 2016 with more than 82% of these injuries classified as mild (http://dvbic.dcoe.mil/dod-worldwide-numbers-tbi, accessed 9December2016). Military personnel who have been concussed may experience persistent cognitive, postural and dynamic stability deficits in addition to disabling headaches, sleep dysfunction, auditory, vestibular, and visual impairments. The occurrence of such symptoms and deficits can limit safe and effective job performance in the inherently demanding military profession [1]. While the vast majority of personnel recover within days or weeks after concussion, for some individuals [2] symptoms can persist impairing duty performance and disrupting a SM’s ability to safely return to duty.

Rehabilitation clinicians currently use a variety of metrics to evaluate acute concussion including subjective symptom reports combined with neuropsychological testing and standardized postural stability assessments such as the Balance Error Scoring System [3]. These approaches, based on a sports medicine model, typically involve comparison of post-injury results to a pre-injury baseline test of the same measures. Pre-injury baseline tests are typically not available for many in the military, so alternatives are needed. Presently, there are limited options for military-based functional assessments to evaluate SM following concussive injury [4]. In deployed settings, Department of Defense (DoD) policy mandates the use of functional assessment measures for Soldiers after three or more concussive events to guide return to duty (RTD) decision-making in that environment [1]. Given the inherent risks associated with premature return to combat [4], this approach acknowledges the importance of assessing complex task performance under realistic conditions. It is noteworthy however, that specific guidance on the type, difficulty and duration of such assessments has not been established, defined or standardized by the DoD or other potential stakeholders in the professional athletics community [1, 4]. To address these gap areas in functional performance testing for military personnel, our military-civilian rehabilitation research team developed a post concussive functional assessment battery to provide RTD guidance following mild traumatic brain injury [4]. The Assessment of Military Multitasking Performance (AMMP) is a battery of functional dual-tasks and multitasks that simulate the combined sensorimotor, cognitive, and exertional demands of common Soldier tasks. The initial AMMP development, which was informed by military stakeholder inquiry, expert consultation and literature review, has been described elsewhere [5]. In brief, the AMMP test battery initially consisted of nine dual-task and multitask assessments which were developed by members of the research team with one investigator serving as the principal developer of each task. Tasks were designed to challenge mild traumatic brain injury (mTBI) related vulnerabilities with deliberate redundancy of the domains and vulnerabilities tested using established dual-task and multitask paradigms [5].

The AMMP purports to measure a complex and heterogeneous concept: “readiness for duty”. Unlike similar functional performance measures however, “readiness to RTD” is not a construct that resides in an isolated performance domain such as physical capability [3, 6–11] or executive function [12, 13]. Common Soldier tasks require decision-making, intact cognitive and sensory function, often in dynamic and stressful environments requiring elite physical abilities. As such, valid measurement of integrated functional performance can be a challenging process relative to standard clinical assessment of isolated performance in physical, sensory or cognitive domains. The AMMP team focused on development of a performance based assessment that targeted known mTBI vulnerabilities. The AMMP had to meet ecological validity standards of military personnel, including commanders, who value the real-world use of the assessment [5]. In addition, practical considerations including the ability to observe and score all task components, administration time, test space requirements, and cost and durability of test materials were all factors that required consideration in the development of the AMMP [5, 10]. Given the consequences of using AMMP metrics to contribute to duty readiness decisions, a vital first step in the AMMP validation process was to determine if the AMMP tasks could be scored reliably.

The purpose of this article is to describe the iterative task refinement process for the Assessment of Military Multitasking Performance including feasibility considerations and the use of repeated interrater reliability testing to improve AMMP tasks, their task metrics, administration, and scoring instructions.

## Methods

This measurement development study involved three sequential phases which used interrater reliability findings, informal and qualitative feedback from raters and subjects, as well as logistical evaluation by the test developers of practical properties to drive task revision. Specifically, the team integrated lessons learned during testing in an iterative manner over successive phases of testing to improve the ability to reliably score task metrics and the quality of subject performance data. Phase I revisions of tasks involved deletion or major revisions to several of the original 9 tasks. For the 6 tasks that remained in the AMMP, Phase II and Phase III revisions involved minor changes such as modifications to operational definitions of metrics or changes to administration instructions for the rater or subject, the type of reporting format used or the addition of a practice trial. Investigators also worked to decrease test burden on participants by decreasing test administration time. Other considerations during task development included consideration of the cost and durability of equipment, and testing space requirements [5]. A research investigator answered all questions and all subjects signed an Institutional Review Board (IRB) approved informed consent as specified by the relevant IRB prior to testing at each phase of AMMP development. Specifically, study protocols were approved by the IRB at the United States Army Research Institute of Environmental Medicine (USARIEM), the Womack Army Medical Center (WAMC) at Fort Bragg, the Allina Health IRB, Minneapolis, MN; and the Quorum Review IRB for Allina Health, Seattle, WA.

Raters for all phases of this study were physical or occupational therapists with between 5 and 31 years of clinical experience. Rater training is described for each phase in the data collection sections below. In summary, training involved individual review of administration and scoring materials, verbal instruction by the principal task developer and practice scoring of 1 or 2 mock subjects. With the exception of the novice rater (AC), all raters participated in the development of one or more of the 6 AMMP tasks.

Apriori, a sample size of 25 was determined to support evaluation of interrater reliability using intraclass correlation coefficients (ICC) where our goal was ICC of 0.9 at a minimum cut point of ICC of 0.7 at a power of 80% and an alpha of 0.05. As a consequence of time and subject availability constraints, we were not consistently able to recruit a full contingent of subjects for the initial reliability trials. However, many of the resulting ICCs were consistent with our theoretical estimations as described in the results section.

### Phase I: Testing in active duty healthy control soldiers

**Fig 1:**
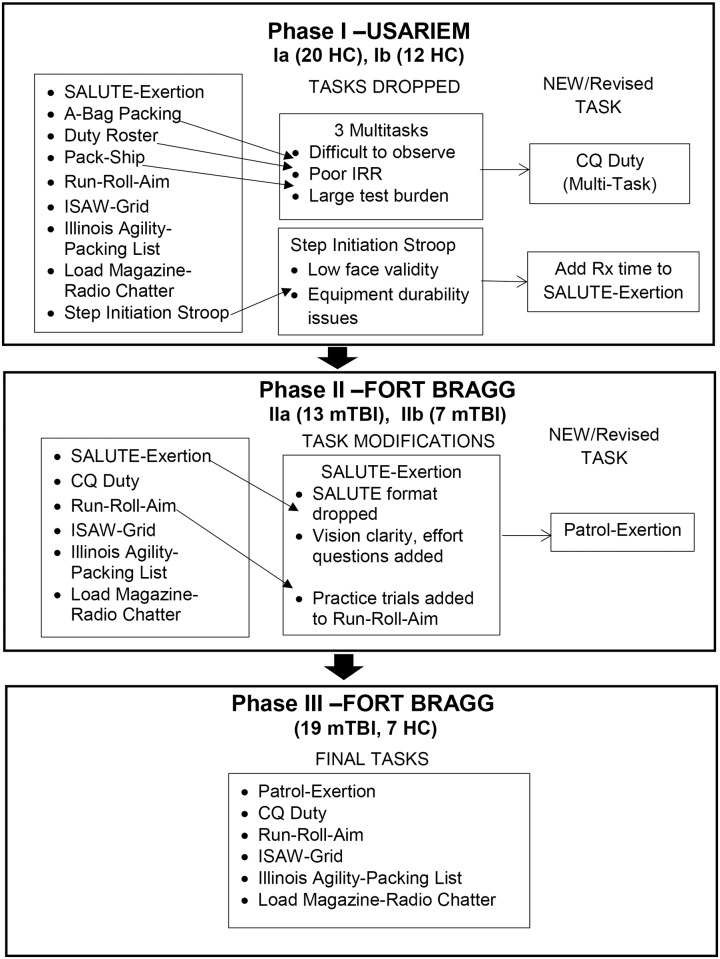
AMMP Testing Phases—iterative task development process. USARIEM = United States Army Research Institute of Environmental Medicine, HC = healthy control, SALUTE = Size, Activity, Location, Unit or Uniform, Time, Equipment; ISAW = Instrumented Stand and Walk, IRR = Interrater reliability, CQ Duty = Charge of Quarters Duty Task, Rx = reaction, mTBI = mild traumatic brain injury.

The goal of this Phase I was to evaluate the interrater reliability (IRR) and feasibility of the AMMP when administered to military healthy control (HC) subjects. There were 2 subcomponents to this phase. During Phase Ia, we evaluated the feasibility and IRR of the original 9 tasks comprising the original version of the AMMP [4, 5]. Three of the original 5 multitasks were eliminated [14] and salient components were refined and consolidated into one multitask called the Charge of Quarters (CQ) Duty Task (Fig 1). The 3 original multitasks demonstrated poor IRR due to unclear operational definitions of success and failure on task components, and difficulty observing all components of each task. One of the 4 original dual-tasks, the Step initiation-Stroop task was dropped after this phase of testing in favor of dual-task assessments with greater face validity [4, 5] and due to concerns about durability of instrumentation. For Phase Ib, feasibility and IRR of 2 tasks were evaluated, the new multitask, CQ Duty, and a revised SALUTE-Exertion multitask which was modified to incorporate improvised explosive device (IED) marker reporting. In a later phase of this study, the SALUTE multitask was revised to incorporate the reaction (Rx) time dimension of the eliminated Step initiation-Stroop task.

#### Phase I-participants

Healthy subjects between the ages of 18 and 42 were recruited by convenience sampling from both human research volunteers (HRV) and permanent party active duty service members from the U.S. Army Research Institute of Environmental Medicine (USARIEM) in Natick, Massachusetts. Participants were excluded if they reported a history of concussion within the previous year or had any residual symptoms from a prior concussion. Subjects were also excluded for 1) documented duty limiting profile for physical or behavioral health condition preventing continuous activity for up to 30 minutes, 2) history of psychiatric disorder and 3) uncorrected hearing or visual deficits preventing functional hearing or vision. In Phase Ia, the initial 9 AMMP tasks were evaluated on 20 healthy control (HC) volunteers (11 males, 9 females, mean age 25.8 (+/-3.5)) with revisions made that required re-evaluation of IRR on 2 of the tasks. In Phase Ib, these two revised tasks (CQ Duty and SALUTE-Exertion) were tested on 12 USARIEM subjects who were a subset of the subjects tested in Phase Ia.

#### Phase I-data collection

To standardize the AMMP task administration for initial IRR evaluation, raters reviewed the task administration instructions, the subject instructions, and the task score sheets on his/her own prior to the beginning of testing. The principal developer for each task provided a brief training session followed by a practice scoring session using teams of 3 raters with a mock subject. The raters then discussed scoring discrepancies and came to consensus on operational definitions for scoring as necessary, which were then included in the guidelines for the task. If necessary, a second mock subject was scored with additional discussion to arrive at a clear consensus among raters. Each scorer then individually rated 20 active duty service member volunteers on each of 4 or 5 of the original 9 tasks. The scorers were both physical (PT) and occupational (OT) therapists (co-authors) with at least one of the raters considered novice or new with limited knowledge or exposure to a particular task. Novice raters had not participated in the development of the task or pilot tested training materials for a given task, thereby providing an unbiased assessment of their experience in rating the task. This iterative approach was used to fine tune task materials and scoring sheets for the tasks over the 3 phases of testing.

During testing, one rater read the scripted instructions and interacted with the subject and all 3 raters scored the subject from direct observation. Primarily during Phase Ia, the AMMP development team provided informal feedback regarding issues with feasibility such as the ability to sufficiently observe a subject complete all components of a task, equipment or software issues, and general practicality regarding ease of administration (i.e., scoring while also following task administration instructions) as well as overall test burden. In Phase Ib, 12 HC volunteers from USARIEM were tested on the revised CQ Duty task and the revised SALUTE-exertion task by a team of 2 OTs and 1 PT.

### Phase II: Testing in active duty soldiers with persistent mTBI related symptoms

The goal of Phase II was to evaluate the IRR and feasibility of the revised AMMP tasks outlined in Table 1 in subjects with mTBI residual symptoms. This phase of testing took place at Fort Bragg, NC with subjects primarily in military occupation specialties related to combat and combat support.

**Table 1:** The Assessment of Military Multitasking Performance tasks, metrics and example modifications.

**Dual-tasks**	**Metrics**	**Description**	**Example Modifications**
**Illinois Agility Test (IAT)-Packing List**	Run time (sec) Words correct Word errors Path errors Dual Task Cost (DTC)	The IAT requires running a course with rapid direction changes and serpentine navigation of 4 cones. In the dual task condition, the subject is given a 5 to 7 word packing list to remember (number of words scaled to number recalled in single task condition).	Instruct raters to stand close to and in front of subject to improve ability to hear call out of recalled “packing list” words. Edit packing list words to remove easily confused (band aid vs bandage) or 3 word items (100 mph tape)
**Radio Chatter-Load Magazine**	Rounds loaded DTC Words Correct Word Errors Words DTC	The subject loads M-16 dummy rounds into a magazine as fast as possible. The dual-task condition requires monitoring radio communication about an upcoming company FTX, responding “check” when key words are spoken by specific characters interacting in the radio chatter.	Instruct subject to “speak loudly so I can hear you”. Instruct raters to sit directly in front of subjects to improve ability to hear the “check” word response by subject.
**Instrumented Stand and Walk-Grid Coordinates (ISAW-Grid)**	Walk time (sec) Grid Coordinates correct DTC time and correct grid coordinates	The ISAW-Grid task involves using wireless wearable inertial sensors and a clinical software program to measure static postural sway and then dynamic stability during walking and turning, the walk is timed. A grid memorization task provided in the context of a patrol mission provides the cognitive challenge.	Instruct subject to “speak loudly so I can hear you”. Instruct raters to stand close to and in front of subject to improve ability to hear call out of recalled grid-coordinates.
**Multitasks**	**Metrics**	**Description**	**Example Modifications**
**Patrol-Exertion (SALUTE format removed)**	IED Markers Correct Patrol Observations correct Visual Clarity (1–10 Visual Analog Scale) Rate of Perceived Exertion (Borg 6–20 scale) Reaction time (msec)	The subject gathers information from video surveillance and radio communications while exercising at 65% to 85% of age predicted maximal heart rate by doing continuous step-ups on an exercise step to simulate a dismounted patrol. IED markers and pertinent logistical information must be recalled and reported at specific times while also requiring a reaction time trigger switch press to an intermittently recurring tone sound (11 tone sounds in 12 minutes).	SALUTE video revised to include IED marker identification to increase task complexity. Addition of auditory reaction time component. Removal of “SALUTE” reporting format with modification to a “question-answer” general PATROL reporting format. Provide specific examples on score sheets as to correct answers to PATROL questions. Addition of symptom report for visual clarity and level of effort (RPE).
**Charge of Quarters (CQ) Duty**	Task Completion Transits Total Task Time (min/sec) Rule Breaks	Requires the subject to organize and implement a plan in order to complete a number of tasks all while pulling CQ duty. Tasks such as assembling a footstool, inventorying supplies, radioing barracks room availability to the 1st Sergeant all occur under time and efficiency rules.	Initial revisions described previously (Smith et al., 2014) Revisions of score sheets to include operational definitions for all task performance metrics and operational definitions of errors. Addition of metrics for several task performance items.
**Run-Roll-Aim (RRA)**	Task completion time (sec) Correct odd/even numbers Total errors: odd/even number and sequence	The RRA task requires the subject to respond to a directional Stroop signal and complete several maneuvers such as avoiding a trip wire, a 3–5 second rush, combat rolls, side shuttling and back pedaling while carrying a simulated weapon. The subject uses a short focal point scope on the weapon to identify numbers on a computer screen based on instructions given before the task starts.	Instruct subject to “call out the numbers loudly so I can hear you”. Instruct raters to stand close to the subject while s/he is calling out numbers to improve ability to hear responses. Improved operational definitions of specific errors (i.e., hesitation). Combination of errors into one metric so all errors were summed.

#### Phase II-participants

A total of 13 SM (12 male, median age 31, range 21–42) with mTBI were recruited from the clinical population receiving rehabilitation services at the Womack Army Medical Center (WAMC) TBI Clinic for persistent symptoms from a concussion occurring from 2 weeks to 2 years (median days (range): 306 (71–470)) prior to the AMMP test date. Physical and occupational therapists from the WAMC Brain Injury Clinic identified potential participants who met the eligibility criteria and provided an information and study contact form. Participants were excluded as described in Phase I. A second set of 7 SM (6 male) were recruited from this same population for additional reliability testing focused primarily on revisions to the SALUTE-exertion task scoring and instructions to subjects.

#### Phase II-data collection

Prior to data collection at Fort Bragg, raters were briefed by each AMMP task developer on changes in test administration or scoring that resulted from prior IRR testing on HC subjects. A practice subject was tested by all raters and any discrepancies in scoring were discussed and clarified by the rater team before subject testing. Three raters were present for this data collection phase that took place over 2 separate test weeks. The first several subjects were consented several days prior to their scheduled testing session. To improve efficiency thereafter, during a single 3 hour session subjects were consented, provided intake information, completed neurocognitive testing and the AMMP. Some subjects were not tested on all AMMP tasks for various reasons including other appointments, duty requirements which took precedence over study participation or poor tolerance to some tasks. Poor tolerance may have involved inability to complete all trials due to symptom exacerbation such as dizziness and headache, or intolerance to white noise that was part of the radio chatter. The tasks that were completed were fully scored by each rater and reliability statistics calculated on completed trials. The order of AMMP task administration varied to minimize a test order effect. Following modifications to tasks based on findings from these subjects, an additional 7 subjects with mTBI (6 male, 1 female) were tested using a different team of 3 raters. These 7 subjects were evaluated on all AMMP tasks, however, the focus of the IRR testing was on further revisions to the SALUTE–Exertion task. After Phase I, most scoring or administrative changes for 5 of the 6 test tasks were minor and primarily involved modifications to operational definitions, improvements to scoresheets or clarifications of instructions to subjects. Following this Phase II, revisions were made to the SALUTE-Exertion task including changes to the reporting format and renaming it the PATROL-Exertion task (Fig 1). Our original intention [5] was to have the rater teams blinded to the subject group for each subject. For practical reasons including recruitment difficulties, subject testing could not be done in larger cohorts that would have allowed raters to be blinded to group.

### Phase III: Mixed cohort testing: HC active duty SM and SM with persistent mTBI related symptoms

The goal of this Phase III was to evaluate the interrater reliability (IRR) of the AMMP tasks after the final revisions to all tasks were completed. Both HC and subjects with mTBI were evaluated in this phase.

#### Phase III-participants

Healthy control subjects were recruited by convenience sampling of volunteers from Fort Bragg Special Operations and the 528 Sustainment Brigade, subjects who responded to recruitment postings placed at fitness centers and cafeterias around the base, as well as volunteers from the Defense and Veterans Brain Injury Center (DVBIC) in-processing briefings. These DVBIC briefings occurred on an almost daily basis as part of the standard informational training provided to all soldiers in-processing to Fort Bragg after transfer from other duty stations. Subjects with mTBI were recruited from the clinical population receiving rehabilitation services at the WAMC TBI Clinic using inclusion criteria as described in Phase II above. A total of 26 subjects were involved in this final phase of IRR evaluation, 7 healthy control (5 male, median (range) age 34 (20–42)) and 19 subjects with residual mTBI symptoms (all male, median (range) age 24 (19–40)). Median days since most recent concussion was 147 (range 63–632).

#### Phase III-data collection

Changes that resulted from Phase II IRR testing were reviewed by task developers with the rater team to standardize AMMP task administration. At this stage, again, most scoring or administrative changes were minor and primarily involved modifications to operational definitions, and improvements to scoresheets. This final phase of data collection for IRR occurred simultaneously with the testing to determine known group validity (which AMMP test tasks could distinguish groups) and for logistical reasons, including proximity to Fort Bragg, involved only 2 raters, both physical therapists. This group of 26 subjects was tested over a several month period usually involving testing one or two subjects in a single day.

### Data analysis for all phases

The Krippendorff Alpha [15] was used to evaluate interrater reliability. This general measure can be used regardless of the number of observers, sample size, missing data and type of measurement (nominal, ordinal, interval, or ratio). For both interval and ratio data the analysis is equivalent to the intraclass correlation coefficient (ICC) for two observers and is extended for many observers. For nominal data, analysis for two observers is equivalent to Scott’s Pi. Parallel analyses using both the Krippendorf and Kappa (2 observers) have produced identical results. The code was integrated into SPSS V18.0. Bootstrapping using an n = 2000 was used to produce 95% confidence intervals. In some cases where the scorers were not constant, the SPSS V18.0 ICC analysis using the two-way random model was used to confirm the Krippendorff result. For items that required a yes/no response, number of triplet (or couplet in Phase III) scoring disagreements was determined. Given that a full range of combinations of responses did not occur, a Kappa-like analysis was not possible. Target ICC was set at >0.90 for dual-tasks and >0.85 for multitasks [16] in order to meet clinical expectations for reliability of assessments.

In addition to the use of IRR findings, both verbal and written feedback from subjects and raters were discussed among the test developers and used to drive task refinement. The first 20 HC subjects tested at USARIEM were administered a team developed “experience survey” which asked for opinions on the clarity of instructions and difficulty of AMMP tasks in addition to requesting general comments on the AMMP test battery. This feedback from test subjects drove edits to clarify test administration instructions and provided impressions of face validity of the individual tasks. Information on administration time for each task was recorded during Phase III testing. Other information on clinical feasibility (test space requirements, cost of equipment) were determined during task development.

## Results (Phases I-III)

The revised AMMP tasks and example modifications are described in Table 1. Each of the 6 tasks uses a unique scoring system (Table 1, left column) related to observable domains and task demands [10]. Three of the tasks also used instrumented measures (inertial sensors) that were not evaluated for interrater reliability and were not modified during this task refinement process.

Table 2 describes demographic characteristics for subjects in each of the three phases of testing. Not all subjects completed all tasks. In order to avoid exceeding the maximum IRB approved test time, subjects who were slower to complete tasks were not asked to begin the last scheduled task. Some subjects did not complete testing due to overlapping scheduling conflicts. Four (Run-Roll-Aim) to 6 (Illinois Agility-Packing List) subjects with mTBI self-selected to stop or were stopped by the primary rater before completing all trials due to an exacerbation of dizziness or headache symptoms. One healthy control subject was stopped from completing all trials of the Illinois Agility Test-Packing List task because he reported a mild aggravation to a prior ankle injury.

**Table 2:** Subject Demographics.

Variable		USARIEM	Fort Bragg	Fort Bragg
		Phase I	Phase II	Phase III
		(N = 20 HC)*	(N = 13 mTBI)	(N = 26, 7 HC, 19 mTBI)
**Age: median (range)**		25 (19–32)	31 (21–42)	25 (19–42)
**Sex**	Female	9 (45%)	1 (7%)	2 (7.7%)
	Male	11 (55%)	12 (92.3%)	24 (92.3%)
**Ethnic background**	Black/African American	8 (40%)	0 (0%)	2 (7.7%)
	Caucasian	7 (35%)	7 (53.8%)	17 (68.4%)
	Hispanic/Latino	3 (15%)	5 (38.5%)	4 (15.4%)
	Other	2 (10%)	1 (7.7%)	3 (11.5%)
**First language: English**		15 (75%)	7 (53.8%)	23 (88.5%)
**Education**	High school	7 (35%)	0 (0%)	6 (23.1%)
	Some college/ Associate degree	6 (54.5%)	7 (53.8%)	15 (57.7%)
	Bachelor’s degree	4 (20%)	5 (38.5%)	4 (15.4%)
	Graduate or professional degree	3 (15%)	1 (7.7%)	1 (3.8%)
**WRAT Reading median (range)** (>12.9 converted to 13)	Standardized	101 (80–134)	89 (83–116)	94.5 (70–134)
	Grade level	12.5 (6–13)	10.2 (6.9–13)	11.2 (3.8–13)
**Years of Service: median (range)**		1.6 (0.3–8.8)	6.8 (2.6–23.0)	3.5 (0.8–18.3)

WRAT = Wide Range Achievement Test- Reading (reading skills and IQ estimate);

*In Phase 1b, CQ Duty and Version 2 of SALUTE-exertion tasks were evaluated on a HC subset, N = 12.

As previously described, findings from Phases I and II informed iterative improvements to the AMMP tasks [5], (Fig 1). After these initial revisions, the remaining 6 test tasks of the AMMP battery continued through this refinement process. Results of the iterative process and phase-specific IRR will be summarized first for the dual-tasks followed by multitasks.

### Dual-task revisions

Illinois Agility-Packing List: Rater reliability for run time and words recalled correctly for the Illinois Agility-Packing List (Table 3) task were > 0.89 for all trials in healthy control subjects tested in Phase I. ICC for packing list errors was poor (0.07 to 0.10) in this HC group. Revisions of scoring rules to require that the recalled word matched exactly the given word (e.g. “bandage” repeated as “band aid” defined as an error) and revision of the initial packing list to replace multiword items (e.g. “100 mph tape”) improved the ICCs for word errors when initially tested on subjects with mTBI (0.54 to 0.85 depending on trial, Phase II) though still below an acceptable range. Further revisions of the packing list ensued, however, the inability of multiple raters to accurately hear responses appeared to contribute to lower reliability. As well, subjects were instructed to speak with sufficient volume and raters were instructed to stand in close proximity to the subject at the end of the agility run. ICC’s for all revised metrics were above 0.96 when tested on the final 23 subjects (Phase III). An additional metric of ‘course error’ was added in order to capture the number of times a subject made an error in navigating the agility course correctly. ICCs for this metric for the final 23 subjects (Phase III) were from 0.77 to 1.0 depending on trial, noting that many subjects made no errors running the agility course.

**Table 3:** Illinois Agility-Packing List Interrater Reliability.

Scoring item (Metrics)	USARIEM*	Fort Bragg/WAMC*	Fort Bragg/WAMC^#^
	Phase I	Phase II	Phase III
	n = 20, Healthy Controls (HC)	n = 12 SM with mTBI	n = 23 (18 mTBI, 5 HC)
	ICC (95% CI)	ICC (95% CI)	ICC (95% CI)
**Single Task Time**	0.98 (0.96–0.99)	0.82 (0.58–0.98)	0.99 (0.98–0.99)
**Single Task Words Correct**	0.89 (0.73–0.99)	0.80 (0.69–0.90)	1.0 (1.0–1.0)
**Single Task Word Errors**	NA	0.54 (0.12–0.83)	1.0 (1.0–1.0)
**Dual Task No Instruction: Time**	0.96 (0.94–0.98)	0.96 (0.93–0.98)	0.99 (0.99–1.0)^@^
**Dual Task NI: Words Correct**	0.93 (0.88–0.97)	0.93 (0.86–0.99)	0.99 (0.97–1.0)
**Dual Task NI: Word Errors**	0.07 (0.0–0.30)	0.93 (0.87–0.97)	0.99 (0.96–1.0)
**Dual Task NI: Course Errors**	NA	NA	1.0 (1.0–1.0)
**Dual Task COG: Time**	0.90 (0.85–0.95)	0.98 (0.97–0.99)	0.99 (0.99–1.0)^@^
**Dual Task COG: Words Correct**	1.0 (1.0–1.0)	0.97 (0.92–1.0)	1.0 (1.0–1.0)
**Dual Task COG: Word Errors**	0.10 (0.0–0.36)	0.74 (0.37–0.99)	1.0 (0.99–1.0)^@^
**Dual Task COG: Course Errors**	NA	NA	0.77 (0.0–1.0)
**Dual Task MOB: Time**	0.96 (0.91–0.98)	0.88 (0.80–0.95)	0.98 (0.97–0.99)
**Dual Task MOB: Words Correct**	1.0 (1.0–1.0)	1.0 (1.0–1.0)	1.0 (1.0–1.0)
**Dual Task MOB: Word Errors**	0.09 (0.0–0.35)	0.85 (0.64–1.0)	0.86 (0.58–1.0)
**Dual Task MOB: Course Errors**	NA	NA	1.0 (1.0–1.0)

COG: Cognitive priority; “concentrate on remembering the words”; NI: no instruction given; MOB: Mobility priority; “concentrate on going as fast as you can”; NA: not assessed;

^@^differences present in 3^rd^ decimal place;

* = 3 raters;

^#^ = 2 raters.

Load the Magazine-Radio Chatter: For this task, all metrics remained unchanged throughout all 3 phases of reliability testing. The ICCs for the Load Magazine-Radio Chatter Dual-task (Table 4) were greater than 0.93 when tested on healthy control subjects in Phase I. When tested on subjects with mTBI (Phase II), the ICCs for distractor words dropped (0.69 in single task, 0.50 in dual task condition). Feedback from raters indicated that in the testing space at Fort Bragg, ambient sounds interfered and not all subjects spoke loud enough to hear over the recorded radio chatter and ambient noise. In addition, responses were sometimes delayed in time after the target word was spoken. Revised instructions to subjects following the first practice trial (if a voice volume issue was identified) were to “speak loudly so we can all hear you” and to require the raters to be seated directly in front of the subject. Clarification of instructions for when a target response was provided in sufficient time to be counted as correct, reduced rater uncertainty for marking a response correct or incorrect. ICCs for the final reliability testing (Phase III) were all greater than 0.95 for this task.

**Table 4:** Load Magazine-Radio Chatter Interrater Reliability.

Scoring item (Metrics)	USARIEM*	Fort Bragg/WAMC*	Fort Bragg/WAMC^#^
	Phase I	Phase II	Phase III
	n = 20 Healthy Controls (HC)	n = 12 SM with mTBI	n = 24 (18 mTBI, 6 HC)
	ICC (95% CI)	ICC (95% CI)	ICC (95% CI)
**Rounds loaded single & dual**^&^	NA	NA	NA
**Correct Key Word Single**	0.99 (0.98–1.0)	0.94 (0.88–0.99)	1.0 (0.99–1.0)^@^
**Distractor Key Word Single**	0.93 (0.83–1.0)	0.69 (0.38–0.92)	1.0 (0.99–1.0)^@^
**Correct Key Word Dual**	0.98 (0.96–1.0)	0.99 (0.97–1.0)	0.98 (0.95–1.0)^@^
**Distractor Key Word Dual**	0.97 (0.95–0.99)	0.50 (0.11–0.82)	0.95 (0.87–1.0)^@^

* = 3 raters;

^#^ = 2 raters;

^&^ = for practical reasons, all dummy rounds were counted one time, not by individual rater; NA: not assessed;

^@^differences present in 3rd decimal place.

Instrumented Stand and Walk-Grid Coordinates: ISAW-grid task metrics (Table 5) remained unchanged throughout all 3 phases of reliability testing. IRR was generally excellent for time and grid coordinate measures (ICCs > 0.92) in HC subjects in Phase I. Initial testing in subjects with mTBI (Phase II) showed a drop in the ICCs (4 were below 0.90) which when later discussed among raters appeared to be the result of some raters not being able to hear the subject vocalize the grid coordinates at the end of the walk and a greater range of error patterns that had not been observed in HC subjects. Changes were made to administration instructions to require raters to move closer in proximity to the subject at the point they finished their walk and to request that subjects speak louder to facilitate hearing their responses over any ambient sounds. Additional clarification of scoring rules and adding the rules to the bottom of the score sheet improved the ability to rate these responses reliably. Final testing on 26 subjects (Phase III) resulted in ICCs > 0.92 for all metrics in the ISAW-grid task.

**Table 5:** Instrumented Stand and Walk-Grid Coordinates (ISAW-grid) Interrater Reliability.

Scoring item (Metrics)	USARIEM*	Fort Bragg/WAMC*	Fort Bragg/WAMC ^#^
	Phase I	Phase II	Phase III
	n = 20 Healthy Controls (HC)	n = 10 SM with mTBI	n = 26 (19 mTBI, 7 HC)
	ICC (95% CI)	ICC (95% CI)	ICC (95% CI)
**Walk Time 1 Single**	0.99 (0.98–1),	0.77 (0.64–0.86)	0.97 (0.96–0.99)
**Walk Time 2 Single**	0.97 (0.96–0.98)	0.95 (0.92–0.98)	0.95 (0.90–0.98)
**Walk Time 3 Single**	0.98 (0.97–0.99)	0.91 (0.85–0.96)	0.98 (0.97–0.99)
**Walk Time 1 Dual**	0.92 (0.85,0.97)	0.89 (0.78–0.98)	0.92 (0.86–0.97)
**Walk Time 2 Dual**	0.98 (0.97,0.99)	0.94 (0.89–0.96)	0.95 (0.90–0.98)
**Walk Time 3 Dual**	0.97 (0.95–0.98)	0.81 (0.72–0.88)	0.98 (0.97–0.99)
**Grid Coord Single**	0.56 (0.14–0.90)	0.88 (0.78–0.97)	0.97 (0.92–1.0)
**Grid Coord 1 Dual**	0.94 (0.90–0.97)	0.94 (0.85–1.0)	0.98 (0.93–1.0)
**Grid Coord 2 Dual**	0.99 (0.97–1.0)	0.99 (0.99–1.0)	1.0 (0.99–1.0)^@^
**Grid Coord 3 Dual**	0.92 (0.84–0.99)	1.0 (1.0–1.0)	1.0 (0.99–1.0)^@^

* = 3 raters;

^#^ = 2 raters;

^@^differences present in 3^rd^ decimal place.

### Multitask revisions

Patrol-Exertion Task: The Patrol-Exertion task underwent multiple revisions in its initial format as a SALUTE report. The SALUTE report is a type of standard Army reconnaissance report which requires specific information related to the size, activity, location, unit or uniform, time and equipment (SALUTE) of the observed enemy. ICCs for various components of the total SALUTE report and the total score ranged from 0.29 to 0.89 in testing 20 HC subjects (Table 6) during Phase Ia and Ib. The addition of the reporting of IED markers seen during scanning reports for Version 2 of the SALUTE tested on 12 HC subjects (Phase Ib) resulted in ICCs that ranged from 0.14 to 0.90. Testing with 7 subjects with mTBI (Phase II) at Fort Bragg was insufficient to calculate ICC, however, disagreements among raters were evident (Table 6). Discussions with subjects, with military subject matter experts, and AMMP developers led to the determination that while reconnaissance reporting is used within the military, actual reporting of intelligence using this SALUTE format tended to be highly variable in degree of detail, ordering, and overall content reported. Those subjects with combat experience were more likely to verbalize their simulated report to “higher command” outside of the standard SALUTE format prioritizing brevity and key findings over the longer, more detailed doctrinal SALUTE format. Reporting format varied greatly between those subjects with mTBI who had been deployed and those that had not. Rank, and previous experience serving in key leadership positions in a patrol also appeared to affect how a Soldier reported pertinent information. Following initial IRR testing at Fort Bragg, the reporting format was changed to a general post-patrol question-answer format with clear criteria for correct and incorrect responses. A reaction time component was added and questions on visual clarity and rate of perceived exertion (RPE) during stepping were also added to the metrics for this task. The ICCs for all metrics for the Patrol-Exertion task were above 0.96 for the final 26 subjects (Phase III) tested at Fort Bragg.

**Table 6:** Patrol-Exertion Interrater Reliability.

SALUTE-EXERTION (V1)		SALUTE-EXERTION (V2)		PATROL-EXERTION	
Scoring item (Metrics)	USARIEM*		Fort Bragg/WAMC*	Scoring item (Metrics)	Fort Bragg/WAMC^#^
	Phase Ia	Phase Ib	Phase II		Phase III
	n = 20 (V1)	n = 12 (V2)	n = 7 SM with mTBI (V2)		n = 26 (19 mTBI, 7 HC)
	ICC (95% CI)				ICC (95% CI)
**Size**	0.85 (0.76–0.93)	0.72 (0.53–0.87)	3 triplets disagreed^1^	X. Sum of IED markers	0.95 (0.91–0.98)
**Activity**	0.29 (0.0–0.60)	0.77 (0.58–0.94)	5 triplets disagreed^1^	Y. Sum of post-test patrol questions	0.97 (0.94–1.0)
**Location**	0.80 (0.64–0.93)	0.78 (0.56–0.95)	1 triplet disagreed^1^	Z. Sum of X and Y	0.97 (0.95–0.99)
**Unit**	NA^@^	0.14 (0.0–0.57)	3 triplets disagreed^1^	Vision clarity initial	0.99 (0.97–1.0)
**Time**	0.57 (0.22–0.86)	0.73 (0.44–0.96)	1 triplet disagreed^1^	Vision clarity end	0.99 (0.98–1.0)
**Equipment**	0.89 (0.79–0.92)	0.81 (0.62–0.95)	3 triplets disagreed^1^	RPE initial	0.98 (0.95–1.0)
**Scan IED Markers**	NA	0.90 (0.76–0.98)	0.97 (0.94–0.99)	RPE end	1.0 (1.0–1.0)
**Total Score**	0.80 (0.66–0.91)	0.79 (0.66–0.90)	0.91 (0.84–0.96)		

^@^ = In the initial version, the report was described as a SALTE report as the “Uniform or Unit” component of the report was not consistently used, based on advice from one early military advisor per local reporting format.

^1^ Due to insufficient n to calculate ICC, the number of rater disagreements is reported. RPE = Rate of Perceived Exertion; IED = Improvised Explosive Device; NA = not assessed;

* = 3 raters;

^#^ = 2 raters;

V1 = Version 1;

V2 = Version 2.

Charge of Quarters Duty Task: ICC findings for CQ Duty (Table 7) for task performance (0.94), number of transits between work stations (0.98) and total task completion time (0.98) were excellent when tested on 12 HC volunteers at USARIEM in Phase Ib. ICC findings for the number of rule breaks was 0.66 in this HC population. ICCs for all metrics (0.90) except rule breaks (0.35) were clinically acceptable when tested on 12 subjects with mTBI (Phase II) at Fort Bragg. Clarifications to operational definitions of rule breaks and inclusion of example rule breaks on the score sheet were some of the revisions made based on these findings and on rater feedback. The ICCs for all metrics for the CQ Duty task were above 0.87 for the final 25 subjects (Phase III) tested at Fort Bragg.

**Table 7:** Charge of Quarters (CQ) Duty Interrater Reliability.

Scoring item (Metrics)	USARIEM *	Fort Bragg/WAMC*	Fort Bragg/WAMC^#^
	Phase Ib	Phase II	Phase III
	n = 12 Healthy Control (HC)	n = 12 SM with mTBI	n = 25 (19 mTBI, 6 HC)
	ICC (95% CI)	ICC (95% CI)	ICC (95% CI)
**Task performance**	0.94 (0.86–0.99)	0.90 (0.84–0.95)	0.88 (0.76–0.97)
**# of Rule breaks**	0.64 (0.32–0.90)	0.46 (0.0–0.79)	0.91 (0.75–1.0)
**# of Visits**	0.98 (0.96–0.99)	0.92 (0.80–0.99)	0.98 (0.97–0.99)
**Total time**	0.98 (0.96–0.99)	0.99 (0.99–1.0)	1.0 (0.99–1.0)^@^

* = 3 raters;

^#^ = 2 raters;

^@^differences present in 3^rd^ decimal place.

Run-Roll-Aim Task: For HC subjects tested at USARIEM in Phase I, the Run-Roll-Aim task metrics demonstrated ICCs of 0.50 to 0.99 (Table 8) depending on the metric. The ability to hear a subject’s verbal identification of visual targets was problematic in the early testing stage, necessitating test instructions to “speak loudly so we can hear you”. This task included a potential error of “hesitation” related to the directional Stroop cue. This element was problematic for rating, given with an incongruent Stroop cue where the letter for right (R) or left (L) roll did not match the directional arrow, some delay is typical, so determining whether a motor delay was of inordinate length was difficult. The individual who is operating the remote that advances the slides that presents the computer cues has an innate sense of the delay post-Stroop presentation that observing raters likely do not. Initial testing in subjects with mTBI (Phase II) continued to demonstrate several reliability coefficients below clinically acceptable levels. Revisions to operational definitions of errors were the primary changes made to this task. Improvement in ICC’s were seen when testing the final 26 subjects at Fort Bragg (Phase III) for time and odd/even numbers (ICC > 0.93) reported. The ICC’s for total errors was 0.64 and for total cues was 0.87 in this group.

**Table 8:** Run-Roll-Aim (RRA) Interrater Reliability.

USARIEM*		Scoring item (Metrics)	Fort Bragg/WAMC	
Phase I			Phase II*	Phase III^#^
n = 20 Healthy Controls (HC)			n = 11 SM mTBI^+^	n = 26 (19 mTBI, 7 HC)
Scoring item	ICC (95% CI)		ICC (95% CI)	ICC (95% CI)
**Trial 1 incongruent numbers correct**	1.0^@^ (0.99–1.0)	Trial 1-Time(secs)	0.91 (0.80–0.99)	1.0 (1.0–1.0)^@^
**Trial 1 incongruent number errors**	2 of 20 triplets disagreed	Trial 1-numbers correct	0.54 (0.08–0.89)	0.96 (0.91–1.0)
**Trial 2 congruent numbers correct**	0.86 (0.65–1.0)	Trial 2-Time (secs)	0.80 (0.57–0.97)	1.0 (0.99–1.0)^@^
**Trial 2 congruent number errors**	2 of 20 disagreed	Trial 2-numbers correct	0.55 (0.0–0.93)	0.93 (0.70–1.0)
**Trial 3 congruent numbers correct**	0.57 (0.15–0.89)	Trial 3-Time(secs)	0.86 (0.67–0.98)	1.0 (0.99–1.0)^@^
**Trial 3 congruent number errors**	2 of 20 disagreed	Trial 3-numbers correct	0.72 (0.40–0.95)	1.0 (0.0–1.0)^@^
**Trial 4 incongruent numbers correct**	0.50 (0.23–0.74)	Trial 4-Time(secs)	0.89 (0.75–0.98)	1.0 (1.0–1.0)^@^
**Trial 4 incongruent number errors**	1 of 20 disagreed	Trial 4-numbers correct	0.99 (0.97–1.0)	0.98 (0.96–1.0)
		Total errors (all trials)	ICC’s for individual trials calculated, T1: 0.54, T2: 0.13,T3: 0.18, T4: 0.85	0.64 (0.13–0.92)
		Total cues (all trials)	ICC’s for individual trials calculated, T1: 0.71, T2: 0.56,T3: 0.37, T4: NA^&^	0.87 (0.66–1.0)

Trial time was not scored by all raters during Phase I.

^@^differences present in 3^rd^ decimal place; Errors for HC recorded as #triplets disagreed as the range of errors was low (0–3 total).

Prior to Phases II & III, revisions were made to score sheets and instructions. Congruent directional Stroop cue = roll direction arrow and R/L letter match; incongruent directional Stroop cue = roll direction arrow and R/L letter do not match. Correct / Errors = odd or even numbers viewed through scope and called out;

^&^ No cues required—all zeros;

* = 3 raters;

^#^ = 2 raters;

^+^ = not all subjects were able to tolerate completion of all trials;

T = Trial.

## Discussion

Rehabilitation assessments that involve multisystem performance often require a multistep process of development and refinement in order to establish IRR [9–11, 14, 15, 17, 18]. Acceptable levels of IRR were achieved for the AMMP dual-task and multitask metrics with the application of a deliberate refinement process that recognized the importance of measure reliability and feasibility. In this early stage of the AMMP validation process, we have chosen to deal with the complexity of the multifaceted metrics that are used in dual-task and in multitask measures, by evaluating IRR for each separate task metric. As the AMMP validation process proceeds, we aim to normalize performance across AMMP tasks, combining individual component metrics to generate a composite score for each task and potentially for the complete AMMP battery. Composite scores should ease interpretation and facilitate decision making as demonstrated with other batteries described in the rehabilitation literature [10, 19].

The AMMP is intended for use in combination with other measures and observations to inform return-to-duty decision-making in SM with mTBI. To make RTD recommendations, the importance of rater reliability in a metric cannot be overstated [16, 20]. Kottner, et. al., suggests that when important decisions on individuals are being made on the basis of an assessment score, rater reliability values should be 0.90 or 0.95 [20]. Not all metrics for the AMMP tasks met this stringent standard however, following the iterative process in the AMMP battery development, the majority of the ICC’s were above 0.90 (Tables 3–8, Phase III), supporting the continuation of the validation process for the component tasks in this assessment battery. Those metrics that did not meet this standard were typically characterized by restricted value ranges which can significantly reduce ICC values.

The process used for refinement of AMMP test tasks began with healthy control (HC) subjects (Phase I). Given that the initial 9 tasks took approximately 3 hours to complete, testing on HC subjects allowed our team to recognize tasks that lacked practicality, feasibility and face validity for healthy active duty SM [5]. Testing in a HC group provided investigators with the early opportunity to evaluate the level of difficulty for individual test tasks among SM considered “duty-ready” and deployable by military standards. Reliability metrics for four of the 6 retained tasks (Tables 3–8) were at or above clinically acceptable levels in this HC group. These findings were consistent with the rehabilitation literature wherein rater reliability for functional tasks are often better (higher) for patients who cluster at the high or the low end of the spectrum ([21],page6).

Phase II testing on subjects with mTBI underscored the importance of optimizing administration and scoring procedures on HC subjects. By the time the AMMP battery was brought to Fort Bragg for testing on subjects with mTBI-related residual symptoms, the mean battery testing time had dropped to 1 hour and 45 minutes, which was reasonably well tolerated by this cohort of participants. With the introduction of subjects with mTBI into the study participant pool, investigators noted a drop in ICCs for several of the AMMP task metrics (Tables 3–8, Phase II) that had previously been at or close to clinically acceptable levels. Subjects endorsing mTBI-related symptoms committed more frequent and wide ranging errors on AMMP tasks than were not observed in testing HC Soldiers at USARIEM. These novel errors required research team members to operationally define rating criteria more explicitly, clarifying acceptable and unacceptable responses. The initial groups of subjects with mTBI also represented a broad range of soldier ranks, occupational specialties and deployment experiences that likely contributed to the variety of participant responses. In addition to the formal test responses, the feedback on the task expectations and realism of the test metrics was made clear to our research team during this process and contributed to practical aspects of task refinement.

Modifications to several AMMP tasks underscored lessons learned and the modifications that were required to achieve clinically acceptable reliability. Development of the IAT-Packing List task highlighted the importance of definitive rules for giving credit for a correct answer. During early reliability testing, some raters gave credit for returned words that were “close but not exact” such as accepting the word “band aid” when the word given was “bandage”. When this “benefit of the doubt” scoring was used by some but not all raters, the IRR suffered. Packing lists were modified to reduce ambiguous or easily misunderstood words. Scoring rules were clarified to require verbatim word responses from the subject, resulting in improved ICCs to clinically acceptable levels. Difficulty with hearing and perceiving items from the packing list occurred more often when test subjects’ first language was not English. Having subjects repeat the words as they are provided could reduce the likelihood of misunderstanding based on accent or language differences of the subject or the tester, but this was not done in this study.

Other real-world lessons learned reinforced the practical requirements for reliable scoring of tests that entail recording subject performance, as well as their verbal responses. Performance-based assessments with verbal responses require appropriate rater vantage point to clearly hear the subject’s responses. If more than 1 rater was scoring, the physical set-up for test administration must be conducive to allow all raters the ability to hear verbal responses. The issue of being able to hear a verbal response may be less of a concern for clinical use of a measure, given the test administrator is typically in the best position to hear a patient’s response and usually is the sole rater of performance.

Development of the Load Magazine-Radio Chatter dual-task and the Patrol-Exertion multitask highlighted the importance of quantifiable metrics that were objective and non-ambiguous requiring no subjective interpretation on the part of the subject or the rater. For example, in the early development phase of the radio chatter, instructions were to respond to chatter that was “relevant” to a specific character in the chatter. Relevance was not sufficiently defined and was interpreted very broadly by subjects during pilot testing. In the final version of this task, participants were instructed to respond affirmatively to the recognition of pre-established words like “break” from specific character voices in the pre-recorded radio chatter which eliminated ambiguity regarding correct and incorrect responses.

Similarly, early versions of the PATROL-Exertion task required reporting using a standard reconnaissance SALUTE report of the sort used during military operations. During several rounds of reliability testing and repeated modifications to subject instructions and scoring examples, answers for this type of reconnaissance report varied and were clearly based on judgment of the subject using their deployment experience, military occupational specialty and rank. Raters also frequently used “benefit of the doubt” scoring depending on the rater’s own experience. Final modifications required post-patrol reporting that had unmistakably defined answers to questions such as “What weapon(s) did you see?” This type of question-response format required focused attention to the Patrol task video without necessitating judgments by the subject or the rater. This change facilitated the clear cut scoring of subject responses by the raters. Sapsford and Jupp discuss issues with observational research including problems with “inconsistency in the way rules are applied by different observers and sometimes by the same observer on different occasions” ([22],page 70). This inconsistency is often seen when inferences for scoring or coding behavior are required and when there are ambiguities in the scoring system [22]. These examples clarify the importance of using well validated tests with clear cut metrics. As well, they underscore the fallibility of make-up-your-own clinical tests involving dual-task and multitask activities and the importance of a standardized test that has established, acceptable rater reliability.

One side benefit of this repeated process of reliability testing and task material revision is the improved and succinct administration instructions for administering the tasks to subjects. Firsthand participation in at least several testing sessions by all members of the AMMP development team helped to maximize the feedback for revisions [10, 11]. Our initial target has been to provide an assessment tool for rehabilitation professionals including physical and occupational therapists. One future goal is to work towards an assessment battery that may be appropriately used by other military medical providers such as nurse practitioners, physician’s assistants, and medics. This goal will require further examination of training requirements and additional reliability testing to compare administration of the AMMP by clinicians who commonly interact with SM to a “gold standard” of trained PT and OT raters. The amount of training required to achieve clinically acceptable reliability has yet to be clarified ([21],page 10).

One of this study’s strengths was the iterative process used that resulted in clinically acceptable interrater reliability that was facilitated by a concise script for test administration and clear, efficient scoring forms. Our final interrater reliability findings (Tables 3–8, Phase III) were sufficient to support continued validation of this assessment tool.

This study has a number of limitations. Despite the defined inclusion criteria, no subjects with mTBI residual symptoms were less than 2 months post most recent concussion. Scoring of and responses from subjects with more acute symptoms and from populations at additional installations or deployment environments, may necessitate further refinement of operational definitions of task metrics. All raters for this study were physical and occupational therapists with a minimum of 5 years of experience and knowledge of the background and development process of the AMMP battery. This may have contributed to a bias in scoring some or all of the AMMP tasks. Further reliability testing with novice raters who did not participate in the development of this assessment will clarify the amount of training required to achieve adequate IRR for a clinical metric. The practical feedback received from the development team and from the subjects resulted in clinically feasible AMMP tasks with some degree of face validity. These tasks, however, may not be feasible in all test environments, as some tasks require a relatively large space or a very quiet test environment without ambient distractions. This will restrict the use of specific AMMP tasks to environments with adequate facilities. The strong face validity and functional relevance likely outweigh the environmental constraints of the testing environments. Use of a clinical utility tool based on DoD needs is premature [23] as no specific directives on which factors (administration time, equipment cost, testing space requirements) have been delineated and likely will vary based on rehabilitation environment. Clinical utility may prove to be a valuable measure in the future once specific test requirements are defined.

Future work to further clarify discriminant and convergent validity with subjects along the continuum of recovery from concussion as well as obtaining comparison or normative data from subjects who are healthy, those with psychiatric issues and those with isolated physical injury will strengthen the value of this assessment in clinical practice. Important next steps will also involve the determination of AMMP responsiveness to change during rehabilitation providing important information to practitioners working with active duty military. Finally, while the current effort with the AMMP battery focuses on RTD decision making in an active duty military population, potential applications include the development and validation of complex tasks to inform return to work standards in civilian operational professions such as police officers or fire fighters. Lessons learned in test development and reliability testing on the AMMP will likely be of great import in the construction of assessment approaches for a broad range tactical athletic professions which draw disproportionally from the ranks of our nation’s veteran population [4].

## Conclusions

Military stakeholder requirements for face validity, and functional relevance contribute to the complexity of development of a reliable AMMP battery as a performance-based assessment evaluating multiple domains of function. The consistency of scores across raters is fundamental to the ability to use the findings of the AMMP to make substantive recommendations regarding duty readiness following concussion/mTBI. Individual AMMP tasks are feasible, and have metrics that can be reliably scored by experienced rehabilitation professionals. Evaluation of preliminary known groups, and convergent validity using correlation to standard neurocognitive tests, is currently underway with members of the AMMP development team. Before the AMMP is used clinically to inform RTD decision-making, further evaluation of intra-rater, novice rater, test-retest reliability, and additional validation studies should be carried out.
